# Haplotype-based genome-wide association study identifies loci and candidate genes for milk yield in Holsteins

**DOI:** 10.1371/journal.pone.0192695

**Published:** 2018-02-15

**Authors:** Zhenliang Chen, Yunqiu Yao, Peipei Ma, Qishan Wang, Yuchun Pan

**Affiliations:** 1 Department of Animal Science, School of Agriculture and Biology, Shanghai Jiao Tong University, Shanghai, PR China; 2 Shanghai Key Laboratory of Veterinary Biotechnology, Shanghai, PR China; China Agricultural University, CHINA

## Abstract

Since milk yield is a highly important economic trait in dairy cattle, the genome-wide association study (GWAS) is vital to explain the genetic architecture underlying milk yield and to perform marker-assisted selection (MAS). In this study, we adopted a haplotype-based empirical Bayesian GWAS to identify the loci and candidate genes for milk yield. A total of 1 092 Holstein cows were sequenced by using the genotyping by genome reducing and sequencing (GGRS) method. After filtering, 164 312 high-confidence SNPs and 13 476 haplotype blocks were identified to use for GWAS. The results indicated that 17 blocks were significantly associated with milk yield. We further identified the nearest gene of each haplotype block and annotated the genes with milk-associated quantitative trait locus (QTL) intervals and ingenuity pathway analysis (IPA) networks. Our analysis showed that four genes, *DLGAP1*, *AP2B1*, *ITPR2* and *THBS4*, have relationships with milk yield, while another three, *ARHGEF4*, *TDRD1* and *KIF19*, were inferred to have potential relationships. Additionally, a network derived from the IPA containing one inferred (*ARHGEF4*) and all four confirmed genes likely regulates milk yield. Our findings add to the understanding of identifying the causal genes underlying milk production traits and could guide follow up studies for further confirmation of the associated genes, pathways and biological networks.

## Introduction

As a highly important trait for breeding, milk yield is directly associated with the economic factors of dairy farming since increased milk yield allows for greater benefits. With the aid of huge advances in marker technology, it is possible for us to dissect heritable quantitative traits such as milk production by mapping the underlying genomic region or quantitative trait locus (QTL). To date, 2 437 QTL intervals correlated with milk yield have been reported on Animal QTLdb for cattle (http://www.animalgenome.org, Release 32, Apr 27, 2017). However, the QTL mapping study traditionally uses a linkage analysis to map QTLs, which results in overlarge intervals that make it difficult to identify the underlying mutation and improve breeding with the use of marker information [1].

With the advent of high-throughput, single-nucleotide polymorphisms (SNPs) genotyping, the genome-wide panels of SNPs allow for a genome-wide association study (GWAS) to explore the genes associated with the complex traits of interest. Compared to the traditional QTL mapping methods, the advantage of GWAS lies in its more precise intervals. Therefore, GWAS has become a widely accepted approach to explore the association between markers and the trait. There are a few GWASs using single-point analysis to identify the key genes for milk yield[2, 3]. For example, Jiang et al. performed a GWAS for milk production traits in a Chinese Holstein population and identified 20 significant genome-wide SNPs for milk yield[2]. However, though GWASs almost always use single-point analysis, the construction of haplotype blocks and identification of tag SNPs are quite informative in the identification of markers [4]. A haplotype analysis with data from a GWAS study proved that it substantially improved the amount of the phenotypic variance explained, compared with single SNPs from a particular region of the genome [5]. Indeed, often neglected as a tool, haplotype-based GWAS may be useful in extracting more information from the dataset and could contribute to the reduction in the missing heritability problem.

Additionally, the most common and efficient model implemented in GWAS is the linear model with the random effect of polygene and fixed effects including marker and population structure such as region, age, etc. However, such models have encountered two issues: the background noise in genomics and the stringency and high rate of false-negatives after Bonferroni correction. Therefore, we adopted a linear mixed model recently developed by our laboratory, and we assumed a haplotype effect as random and to be normally distributed [6]. By using an empirical Bayesian (EB) method, the prior variance is the estimate from the same dataset, and the posterior mean is the best linear unbiased prediction (BLUP) of the marker effect. The present study conducted a haplotype-based GWAS with an empirical Bayesian method for milk yield traits in Shanghai Holsteins. We tried to analyze the blocks with 2, 3 and 4 SNPs, find the significant blocks, and identify the associated genes, pathways and networks important for the milk production trait to guide the improvement of dairy cattle breeding.

## Material and methods

### Population and phenotypes

Approval by the Institutional Animal Care and Use Committee of Shanghai Jiao Tong University (contract no. 2015-07-0136) was given for all experimental procedures involving animals in the present study. A total of 1 092 cows were selected from 24 farms in Shanghai Bright Holstan Co., Ltd., with the following criteria: 1) primiparous cows born between 2001 and 2012 with the regular and standard performance of DHI (milk yield, fat percentage, protein percentage and somatic cell count); 2) age at first calving between 24 months and 36 months; and 3) test day from 5 to 335 DIM. The blood samples were collected along with regular quarantine inspection of the farms. The estimated breeding values (EBVs) were used as phenotypes in this study. EBVs were calculated by using a random regression test-day model with fixed effects of herd test day and fixed regression coefficients, which differ by season of calving, and random regression coefficients for additive animal and permanent environment. The modified Wilmink function [7] described in [8] was modeled as a covariate for both fixed regression and random regression. Variance component analyses and the estimation of EBVs were run using the BLUPF90 software [9].

### Genomic data

A total of 164 312 high-confidence SNPs with minor allele frequencies (MAFs) ≥ 0.05 were detected by using the GGRS method[10]. Briefly, the DNA was extracted from blood samples, and all 1 092 Holstein cows were sequenced. The raw reads with a base average quality score of at least 20 (error rate of base-calling of 1 in 100) and of at least 30 (error rate of base-calling of 1 in 1000) in the first 65 bp aligned to the cow reference genome were retained. The filtered reads were aligned to the UMD3.1 assembly of the cattle genome [11] by using the Burrows-Wheeler Aligner (BWA) [12]. The successfully aligned reads were used to discover SNPs by using SAMtools software. These SNPs were retained for further analysis based on the following criteria: more than 30% genotyped samples and sequencing depth greater than 5-fold on average. Eventually, the missing genotypes were imputed by iBLUP [13]. The SNP and phenotype data are freely available at public repository Dryad (https://doi.org/10.5061/dryad.cs133). The haplotype phase was inferred with the BEAGLE v4.1 software (Browning et al., 2007). Haplotype blocks in 1 092 Holstein cows were detected using PLINK v1.07 software for each chromosome using the method proposed by Gabriel et al. [14]. Haplotype blocks containing two SNPs, three SNPs and four SNPs were used to perform GWAS by using the following statistical model.

### Statistical model

We adopted a haplotype-based empirical Bayesian model inherited from a SNP-based method proposed by our group [6]. Here, we use a block with 2 SNPs as an example to demonstrate the theory and methods. The method holds for blocks with any SNPs. Let *y* be an *n* × 1 vector of phenotypic values for *n* individuals. Define *Z*_*k*_ as an *n* × 4 matrix of haplotype inheritance for block *k*. The *j*th row of matrix *Z*_*k*_ is defined as a 1 × 4 vector. If this individual carries the first and second haplotypes, then

$$Z_{jk}=\left[1\;1\;0\;0\right]$$

If the individual is a homozygote with the third haplotype, then *Z*_*jk*_ is defined as

$$Z_{jk}=\left[0\;0\;2\;0\right]$$

The general rule for defining *Z*_*jk*_ is that there are at most two non-zero elements, and the sum of all four elements equals two. Let

$$\gamma_{k}={\left[\gamma_{1k}\;\gamma_{2k}\;\gamma_{3k}\;\gamma_{4k}\right]}^{T}$$

Let *k* be the kth haplotype block under consideration. The model is

$$y=X\beta +Z_{k}\gamma_{k}+\xi +\varepsilon$$

The variance matrix of *y* is

$$\mathrm{var}(y|X,\beta )=(Z_{k}Z_{k}^{T}\lambda_{k}+K\lambda +I)\sigma^{2}$$

where *λ* = *ϕ*^2^/*σ*^2^ and $\lambda_{k}=\phi_{k}^{2}/\sigma^{2}$.

Eigen decomposition can be used to save the computation time of estimating multiple genetic variance components.

The eigen-decomposition for matrix *K* is *K* = *U D U*^*T*^. Let y^*^ = *U*^*T*^*y*, x^*^ = *U*^*T*^*x* and $Z_{k}^{*}=U^{T}Z_{k}$ represent the transformed variables, so that

$$y^{*}=X^{*}\beta +Z_{k}^{*}\gamma_{k}+U^{T}(\xi +\varepsilon )$$

The variance-covariance matrix of y^*^ is

$$\mathrm{var}(y^{*}|X^{*},\beta )=(Z_{k}^{*}Z_{k}^{*T}\lambda_{k}+R)\sigma^{2}=H_{k}\sigma^{2}$$

where *R* = *Dλ* + *I* is a diagonal matrix, and $H_{k}=Z_{k}^{*}Z_{k}^{*T}\lambda_{k}+R$ is a general covariance structure. After eliminating the parameters *β* and *σ*^2^, we have the following profiled restricted log-likelihood function:

$$L(\lambda_{k},\lambda )=-\frac{1}{2}\ln|H_{k}|-\frac{1}{2}\ln|X^{*T}H_{k}^{-1}X^{*}|-\frac{n-r}{2}\ln(y^{*T}P_{k}y^{*})$$

where

$$P_{k}=H_{k}^{-1}-H_{k}^{-1}X^{*}{\left(X^{*T}H_{k}^{-1}X^{*}\right)}^{-1}X^{*T}H_{k}^{-1}$$

This likelihood function contains only two parameters: *θ* = {*λ*_*k*_,*λ*}. The Newton-Raphson algorithm can be used to calculate the numeric solution of *θ*. We can then obtain the empirical Bayesian estimate of haplotype effects and construct a Wald test statistic. Assume that the Wald test statistic follows a Chi-square distribution with one degree of freedom. The p-value is calculated using $p_{k}=\mathrm{Pr}(\chi_{1}^{2}>W_{k})$.

### QTL data collection and pre-processing

Cattle QTL data were downloaded from the animal QTL database (http://www.animalgenome.org/, Release 32, Apr 27, 2017). Based on both the QTLs associated with milk in cattle and the genome's location information, we could obtain the initial gene set associated with the milk-related QTLs by using a brief Perl script. After defining the initial gene set, we performed multi-level bioinformatics analyses to explore the potential biologically significant genes harbored in the QTL regions.

### Identifying bovine milk trait function gene sets by ingenuity pathway analysis (IPA)

We use the IPA to filter the prioritized genes and quickly visualize their regulatory networks by their specific relationships with milk-associated biological pathways. As research with IPA is mostly on the human and the rat, we use human or rat homologous genes of the uploaded gene set to perform the analysis in which we are searching for the genes associated with milk yield. The analysis returns gene sets and associated networks based on the IPA database and gene function. We select the network with the most relevant milk-associated genes in the uploaded set, and we infer that the rest of the genes in the network from the uploaded gene set may have potential relationships with milk yield.

## Results

Across all 13 476 tested blocks, we found a total of 17 blocks whose associations with milk yield were statistically significant, produced three Manhattan plots, identified the associated genes and annotated them with the milk-associated QTL intervals and IPA networks. It should be noted that the 17 blocks were found when using a modified Bonferroni (mBon) correction, compared with only 10 blocks that could be identified by using a classical Bonferroni correction. The EB method, the use of an ‘effective number of tests’ rather than the ‘total number of markers’ to correct for multiple tests allowed for a reduced p-value threshold from 0.05/13 476 = 3.71 × 10^−6^ (classical Bonferroni correction) to 0.05/618.31 = 8.09 × 10^−5^ under the modified Bonferroni correction. Among the 17 significant blocks, as presented in S1 Table, 9 were from the blocks with 2 SNPs, and another 8 came from the blocks containing 3 SNPs. No significant block was found among the blocks with 4 SNPs.

**Fig 1:**
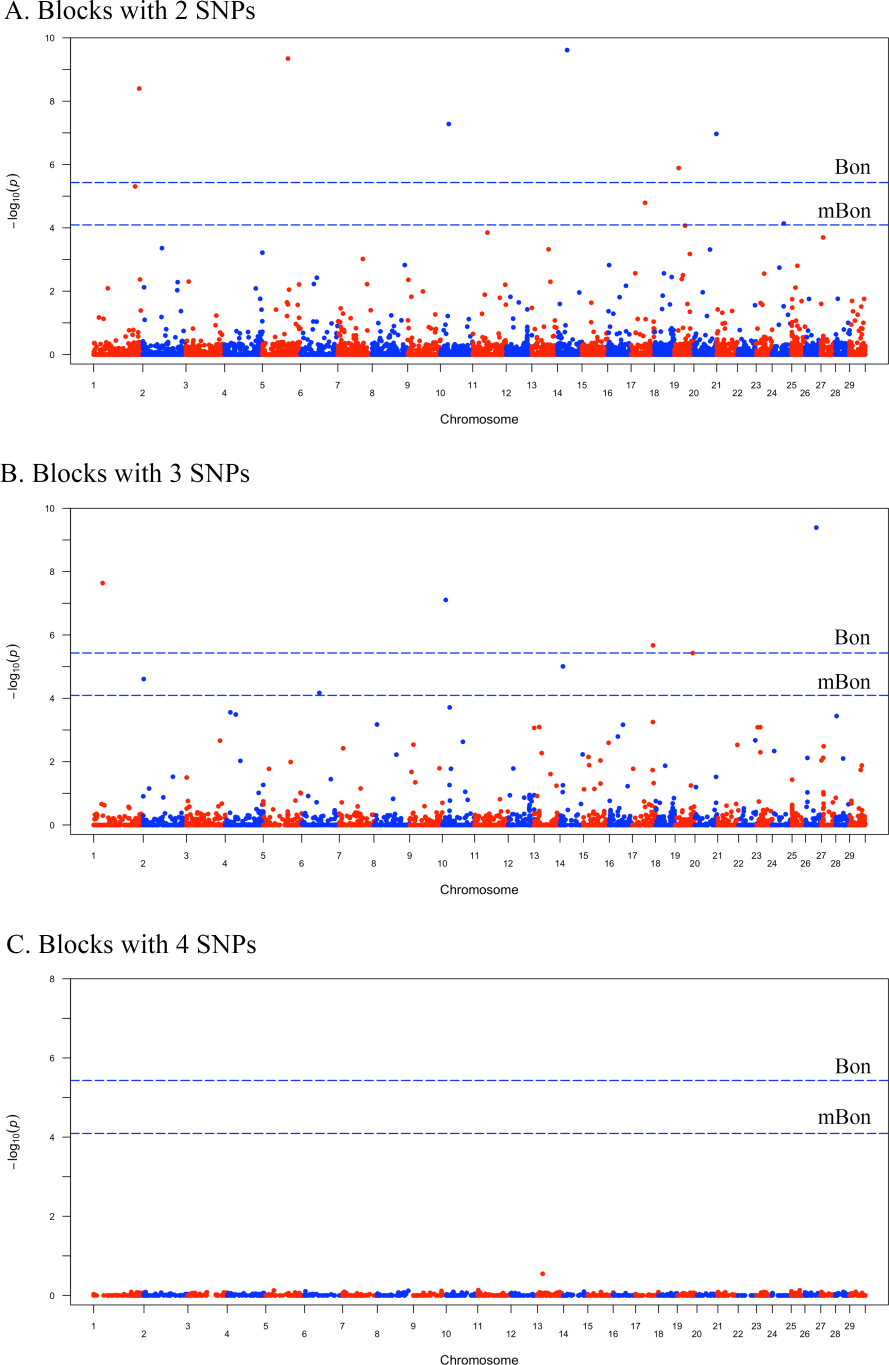
Manhattan plot for blocks with 2, 3, and 4 SNPs. The upper line is the threshold obtained from a classical Bonferroni correction, with a P-value of 8.09E-05, and the lower line is the threshold of a modified Bonferroni-corrected threshold, with a P-value of 3.71E-06. The number of significant blocks detected by using a classical Bonferroni (Bon) on Fig 1(A) was 6, while the modified Bonferroni (mBon) could detect 3 more blocks. Similarly, Fig 1(B) shows that only 4 blocks were detected by using a classical Bonferroni, but the modified Bonferroni could identify 8 significant blocks. No block was significant in the blocks with 4 SNPs, as shown in Fig 1(C).

According to the number of SNPs contained in blocks, three Manhattan plots (Fig 1) were produced to display the profiles of the P-values (in terms of -log(p)) of tested blocks. The two threshold lines were calculated based on a classical Bonferroni (Bon) and a modified Bonferroni (mBon) correction. In a total of 11 of the 17 significant blocks, the associated genes could be found within the interval, while the other 6 blocks contained no genes. A total of 24 genes were located within 50 kb upstream and downstream of the block region. For each SNP, we selected and listed the closest gene (11 genes in all). Of all 27 SNPs in the blocks containing genes, 13 were within the associated genes of the blocks; others were located less than 20 kbp away from the nearest gene. Four blocks, with the genes *ITPR2*, *OR4N5*, *THBS4* and *TDRD1*, were highly significant, with a P-value reaching the level of 10^−8^. The details of the significant blocks, corresponding SNPs, nearest known genes and P-values are presented in Table 1. We have also performed single-locus GWAS to compare with the haplotype-based GWAS, and only three SNPs within Block 2–2, Block 2–8 and Block 3–5 were identified after Bonferroni correction.

**Table 1:** Genome-wide significant blocks for milk yield.

Block	Chr.	Bonferroni correction	SNPs	Nearest Gene		P-value
				Name	Distance	
Block 2–1	1	Only mBon	135 886 514	CEP63	Within	4.93E-06
			135 886 546	CEP63	Within	
Block 2–2	5	Both Bon & mBon	83 678 733	ITPR2	Within	4.51E-10
			83 678 739	ITPR2	Within	
Block 2–3	10	Both Bon & mBon	27 514 423	OR4N5	9 432	5.28E-08
			27 514 460	OR4N5	9 395	
Block 2–4	17	Only mBon	45 596 775	FBRSL1	17 085	1.62E-05
			45 596 988	FBRSL1	16 872	
Block 2–5	19	Both Bon & mBon	15 017 221	AP2B1	9 149	1.29E-06
			15 017 461	AP2B1	8 909	
Block 2–6	24	Only mBon	38 309 144	DLGAP1	15 261	7.31E-05
			38 309 195	DLGAP1	15 312	
Block 3–1	2	Only mBon	1 590 666	ARHGEF4	18 375	2.47E-05
			1 590 672	ARHGEF4	18 369	
			1 590 684	ARHGEF4	18 357	
Block 3–2	10	Both Bon & mBon	10 935 068	THBS4	10 357	7.84E-08
			10 935 325	THBS4	10 100	
			10 935 342	THBS4	10 083	
Block 3–3	14	Only mBon	10 142 741	OC90	Within	9.83E-06
			10 142 746	OC90	Within	
			10 142 975	OC90	Within	
Block 3–4	19	Only mBon	57 737 460	KIF19	Within	3.74E-06
			57 737 480	KIF19	Within	
			57 737 777	KIF19	Within	
Block 3–5	26	Both Bon & mBon	34 961 904	TDRD1	Within	4.06E-10
			34 961 905	TDRD1	Within	
			34 961 908	TDRD1	Within	
Block 2–7	1	Both Bon & mBon	149 340 609			4.01E-09
			149 340 632			
Block 2–8	14	Both Bon & mBon	31 436 218			2.44E-10
			31 436 401			
Block 2–9	20	Both Bon & mBon	74 328 375			1.08E-07
			74 328 383			
Block 3–6	1	Both Bon & mBon	29 116 305			2.30E-08
			29 116 327			
			29 116 348			
Block 3–7	6	Only mBon	57 444 519			6.78E-05
			57 444 521			
			57 444 587			
Block 3–8	17	Both Bon & mBon	66 974 027			2.13E-06
			66 974 043			
			66 974 064			

To further annotate the significant blocks, we compared the block intervals with the QTL regions associated with milk. As shown in Table 2, seven blocks were located within the milk-related QTLs. Among the 7 blocks, two blocks, Block 3–6 and Block 3–8, did not harbor any gene but were within the reported milk-associated interval on QTLdb. The closest genes associated with the other five blocks within the QTL intervals were *CEP63*, *ITPR2*, *THBS4*, *KIF19* and *TDRD1*. All 7 of the blocks were correlated with certain substances in milk. Two blocks located on Chr. 1 showed relationships with the chemical elements zinc and phosphorus, while the other five were linked to acid percentage in milk, including myristic acid, capric acid and caprylic acid. By calculating the haplotype frequencies for each block, we found that the dominant haplotype for the two 2-SNP blocks was “H00”, while the remaining 5 blocks with three SNPs were dominated by different haplotypes, including “H011”, “H110”, “H111”, and “H000” (S2 Table).

**Table 2:** Milk-associated QTL intervals for significant blocks.

Blocks	Associated gene	Chr.	QTL Interval		Description
			Start	End	
Block 3–6	Not found	1	5 541 350	62 148 459	Milk zinc content
Block 2–1	CEP63	1	44 984 520	145 633 241	Milk phosphorus content
Block 2–2	ITPR2	5	76 533 399	93 514 025	Milk myristic acid percentage
Block 3–2	THBS4	10	10 139 639	11 156 367	Milk capric acid percentage
Block 3–8	Not found	17	48 057 944	67 505 450	Milk myristoleic acid percentage
Block 3–4	KIF19	19	36 754 043	61 016 756	Milk caprylic acid percentage
Block 3–5	TDRD1	26	1 419 676	38 996 499	Milk capric acid percentage

**Fig 2:**
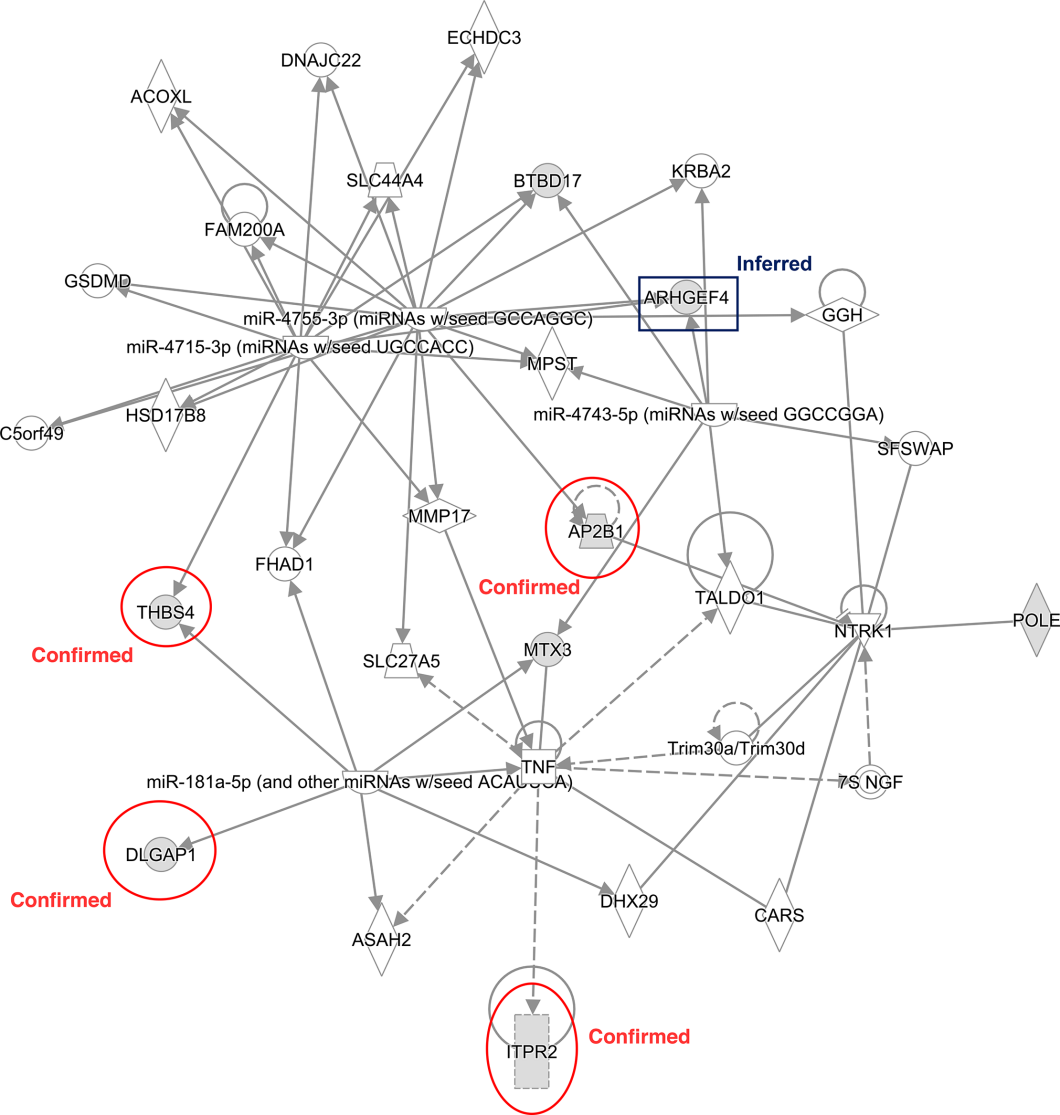
IPA network for genes associated with the significant blocks. Biological network of the associated genes within the significant blocks, with the solid lines indicating direct interactions and dashed lines indicating indirect interactions. The eight genes marked in gray, *AP2B1*, *ARHGEF4*, *BTBD17*, *DLGAP1*, *ITPR2*, *MTX3*, *POLE* and *THBS4*, are those involved in the submitted list. Among them, 4 genes (*AP2B1*, *DLGAP1*, *ITPR2*, and *THBS4*) are confirmed, and 1 gene (*ARHGEF4*) is inferred to be associated with milk yield from previous reports and QTL analysis.

Additionally, we submitted a dataset containing all 24 genes detected in 11 blocks to IPA software, and we obtained the network analysis shown in Fig 2. The network involved 35 molecules in all, and eight of them were genes in our submitted dataset, namely, *AP2B1*, *ARHGEF4*, *BTBD17*, *DLGAP1*, *ITPR2*, *MTX3*, *POLE* and *THBS4*. Among them, five genes, *AP2B1*, *ARHGEF4*, *DLGAP1*, *ITPR2* and *THBS4*, were the nearest genes to the corresponding SNPs in the blocks, as listed in Table 1. The other three, *BTBD17*, *MTX3* and *POLE*, were located approximately 15 kbp, 35 kbp, and 25 kbp away from the corresponding SNPs, respectively. The score of the network was 19, and the biological processes defined by IPA were related to lipid metabolism. In addition, we identified the 7 blocks (Blocks 2–2, 2–4, 2–5, 2–6, 3–1, 3–2, and 3–4) harboring these 8 genes, and we compared the dominant haplotypes for each block. From S2 Table, the frequency of “H00” for all four blocks with 2 SNPs was the highest, but for three 3-SNP blocks, the dominant haplotype varied. Blocks 3–1, 3–2, and 3–4 were dominated by “H000”, “H011”, and “H110” with frequencies of 0.776, 0.905 and 0.819, respectively.

Finally, we calculated the allele frequencies of each SNP contained in 17 significant blocks to display the current population, and the MAFs are listed in S3 Table. There were 42 SNPs in all, most of which had a low or medium frequency of their minor alleles. Twenty SNPs had an MAF under 0.1, and another 18 SNPs’ MAFs were larger than 0.1 but less than 0.3. Only 4 SNPs displayed a high frequency of their minor alleles (larger than 0.3). Among the 4 SNPs, three were located on Chr. 17, and the other one was located on Chr. 24. All the SNPs on Chr. 10, Chr. 20, and Chr. 26 had an MAF lower than 0.1, while all MAFs of the SNPs on Chr. 2, Chr. 5, and Chr. 6 were within the interval of 0.3–0.5. Additionally, we compared the contributions of the major alleles and minor alleles to milk yield. It turned out that except for two SNPs on Chr. 20, all the other 40 major alleles were the favorite genes in the current population.

## Discussion

In sum, we performed a genome-wide association study based on haplotypes to identify the loci and correlated genes responsible for milk yield traits in Shanghai Holsteins. To our knowledge, it is the first GWAS for milk production traits using a haplotype-based empirical Bayesian model. Employing the empirical Bayesian method proposed by Wang et al., the model treated the effect of a haplotype as a random variable and assumed it to be normally distributed [6]. The prior variance under EB theory of the marker effect of interest could be estimated from the data. In addition, the EB method allowed us to use the ‘effective number of tests’ rather than ‘total number of markers’ to perform a modified Bonferroni correction, resulting in a considerable decrease in the threshold of P-values. Compared with classical Bonferroni correction, we obtained 7 more significant blocks, and interestingly, two corresponding genes, *DLGAP1* and *ARHGEF4*, might have relationships with milk production traits and will be discussed later in detail. Finally, we detected 17 blocks in all that appeared to be significantly related to milk production traits in Shanghai Holsteins.

Considering that the closest gene to each SNP in the block could provide more accurate information, we based our analysis of genes mainly on the nearest genes, as listed in Table 1. Of the 11 nearest genes for 27 significant SNPs, four showed convincing associations with milk yield traits in previous reports, namely, *DLGAP1*, *AP2B1*, *ITPR2* and *THBS4*, on Chr. 24, Chr. 19, Chr. 5 and Chr. 10, respectively. First, *DLGAP1* has been identified as a significant gene associated with milk yield based on the genomic analysis of 15 745 SNPs in buffaloes that was performed to find those associated with milk yield and content [15]. The P-value of *DLGAP1* in our study was 7.31 × 10^−5^, and it was only identified when using our modified Bonferroni (mBon) correction, which proves the effectiveness of the EB-mBon method. For another two genes, *AP2B1* and *ITPR2*, Kolbehdari et al. performed a whole-genome scan to identify the QTLs affecting milk production traits using 1 536 SNP markers [16]. The results showed that the genes *AP2B1* and *ITPR2* were associated with four significant SNPs related to the persistency of milk yield and fat yield in milk. In addition, our result suggested that *ITPR2* was a highly significant gene with a P-value of 4.51 × 10^−10^, and it was also detected to be located within a QTL region with a relationship to myristic acid percentage in milk. The *ITPR2* gene also has been reported to be associated with fat percentage in previous GWA studies [2, 17]. The last gene, *THBS4*, has been recognized as a differentially expressed gene between the mammary gland of two groups of cows with extremely high and low milk protein percentage and fat percentage by Cui’s investigation of the complexity of the mammary gland transcriptome in dairy cattle using RNA-seq[18]. In our study, *THBS4* was also located on the QTL interval associated with capric acid percentage in milk. Therefore, four of the 11 significant nearest genes detected by using our EB-mBon method (*DLGAP1*, *AP2B1*, *ITPR2* and *THBS4*) could be confirmed to have definite correlations with the traits of interest, which demonstrates the validity and practicability of our method.

Another three genes, *ARHGEF4*, *TDRD1* and *KIF19*, were not reported to be directly linked to milk production traits. However, they participate in relevant biological processes, which might have certain associations with milk yield. *ARHGEF4* was identified to be involved in two networks of bovine milk proteins, which means that it is likely to be a factor influencing the proteins in milk [19]. In addition, Cremonesi conducted a meta-analysis that combined six independent studies of infected mammary glands to identify the differentially expressed genes, and *ARHGEF4* was one of them and was placed into a pathway associated with phospholipase C signaling [20]. The annotations from Gene Ontology (GO) also showed its function of protein binding and regulation of protein signal transduction. Thus, it is reasonable to presume that *ARHGEF4* is highly likely to affect milk production traits. Also worth mentioning is that *ARHGEF4* is a gene other than *DLGAP1* that was only detected under our modified Bonferroni correction. This again proved that the EB-mBon method could greatly increase statistical power and identify more genes associated with the trait of interest. The gene *TDRD1* was annotated with germ cell development and DNA methylation involved in gamete generation on GO. Additionally, Chitwood performed an RNA-seq analysis of single bovine blastocysts and discovered that *TDRD1* was overexpressed in embryos and was involved in the biological process of the negative regulation of gene expression [21]. In our study, the P-value for the corresponding block of the gene *TDRD1* reached the level of 10^−10^, showing extremely high significance. Finally, the gene *KIF19* was also reported to be involved in a network associated with inflammatory disease and response in mouse mammary glands during lactation [22]. In addition, both the genes *TDRD1* and *KIF19* are within the QTL interval, representing the relationship to capric acid and caprylic acid percentage in milk, respectively. Therefore, it could be inferred that *ARHGEF4*, *TDRD1* and *KIF19* might exert some influence on milk yield traits. Additional research and experiments are needed to confirm these genes’ relationship to milk yield and guide the breeding of Shanghai Holsteins.

Another 4 genes (*OC90*, *CEP63*, *FBRSL1* and *OR4N5*) have few reports regarding their association with milk yield traits, but they were involved in multiple biological processes and molecular functions. *OC90* is a novel gene located on Chr. 14, and it participates in several metabolic and catabolic processes as well as the regulation of molecular activities. *CEP63* is a centrosome protein contributing to chromosomal stability by preventing centrosome overduplication [23]. Although *CEP63* is located in a QTL region with milk association, so far, there is no convincing evidence to prove that it is responsible for milk production traits. *FBRSL1* is fibrosin-1-like and crucial for many biological processes in mammals, including stem cell maintenance and differentiation [24]. *OR4N5* is an olfactory receptor gene involved in multiple signaling pathways and receptor activities. Further studies are needed to deeply explore their associations with milk yield and then determine whether the four genes are real factors or are just false positives. The relevant references and conclusions for all 11 genes are summarized in Table 3. The biological networks for the submitted genes in IPA show satisfactory results as well, which could help explain the relationships of both the single-gene and integrated networks to milk production traits. It is so exciting to determine that all 4 previously confirmed genes, *DLGAP1*, *AP2B1*, *ITPR2* and *THBS4*, as well as one inferred gene, *ARHGEF4*, are placed in this network. Therefore, it is possible that the network participates in the regulation of milk yield, although it has not yet been annotated by IPA. Meanwhile, the remaining 3 genes in the network, *BTBD17*, *MTX3* and *POLE*, might also be implicated in the regulation of milk yield by performing certain biological functions. More research is needed to explore whether and how the network is closely linked to milk production traits.

**Table 3:** Summary of the significant genes.

Chr.	Gene	P-value	References	Conclusion
24	DLGAP1	7.31E-05	Venturini, Cardoso et al. (2014)	Confirmed
19	AP2B1	1.29E-06	Kolbehdari, Wang et al. (2009)	Confirmed
5	ITPR2	4.51E-10	Kolbehdari, Wang et al. (2009)	Confirmed
10	THBS4	7.84E-08	Cui, Hou et al. (2014)	Confirmed
2	ARHGEF4	2.47E-05	D'Alessandro, Zolla et al. (2011)	Inferred
26	TDRD1	4.06E-10	Chitwood, Rincon et al. (2013)	Inferred
19	KIF19	3.74E-06	Le Guillou, Sdassi et al. (2012)	Inferred
1	CEP63	4.93E-06	He, Zhao et al. (2015)	Certain biological process
17	FBRSL1	1.62E-05	Bathla, Rawat et al. (2015)	Certain biological process
10	OR4N5	5.28E-08	Mauer (2011)	Certain biological process

Additionally, we calculated the minor allele frequencies (MAF) of the 42 SNPs contained in 17 significant blocks and evaluated the average allele effects for milk yield. Most of the SNPs displayed a frequency lower than 0.3, and the result shows that 40 SNPs in the tested population were dominated by favorite genes with larger contributions to milk yield. On only two SNPs, located at 74 328 375 and 74 328 383 bp on Chr. 20, the favorite genes had lower allele frequencies. Further validation of these two SNPs in multiple populations is needed, and if they do affect the milk yield, this could be applied to the improvement of breeding strategies. To increase the milk yield, we could select the samples with the two SNPs dominated by favorite genes and thus increase the frequency of favorite genes in the population. For those with lower frequencies of favorite genes on the two SNPs, we could insert the desired gene artificially by using genetic engineering.

Compared to several previously reported GWA studies using the Illumina BovineSNP50 BeadChip, the SNPs were identified by using a reduced sequencing method [10] in the present study. Approximately thirty thousand SNPs were overlapping between the two SNP-detection platforms, and some SNPs located in known genes were not detected by our platform. For example, one famous DGAT1 gene with large effects on milk traits in Holstein cows has been reported by several GWA studies. However, no SNPs within DGAT1 were identified in the present study.

In conclusion, we identified 17 significant blocks in all, and in 11 of them, genes could be found within the block interval. Of the 24 genes within the blocks, we focused the analysis on the 11 genes nearest to the SNPs in blocks. From QTL analysis and previous reports, we confirmed that *DLGAP1*, *AP2B1*, *ITPR2* and *THBS4* do have certain relationships with milk yield traits, while three genes, *ARHGEF4*, *TDRD1* and *KIF19*, could be inferred to be associated with milk production. Additionally, a biological network containing all four confirmed genes and one inferred gene might participate in the regulation of milk yield. Further studies on the inferred genes, pathways and biological networks across multiple populations should be conducted to confirm their roles in milk production. We believe that our findings provide new insights into the exploration of the genes responsible for milk production traits, and they could guide the improvement of the breeding systems for Shanghai Holstein and other dairy cattle.

## Supporting information

S1 Table Number of significant blocks detected by using different corrections. (DOCX)

S2 Table Haplotype frequencies for each block. (DOCX)

S3 Table MAF of each SNP in significant blocks. (DOCX)
