# Supplementary material for: Haplotype-based genome-wide association study identifies loci and candidate genes for milk yield in Holsteins
Source: PLoS One. 2018 Feb 15;13(2):e0192695. doi: 10.1371/journal.pone.0192695 (PMC5813974; doi:10.1371/journal.pone.0192695)
Supplement: S1 Table — (DOCX) [file pone.0192695.s001.docx]

S1 Table. Number of significant blocks detected by using different corrections

| No. SNP | Tested blocks | effective number | mBon | Bon |
| --- | --- | --- | --- | --- |
| 2 | 8960 | 477.58 | 9 | 6 |
| 3 | 3256 | 125.43 | 8 | 4 |
| 4 | 1260 | 15.3 | 0 | 0 |
| All | 13476 | 618.31 | 17 | 10 |

^a^The modified Bonferroni-corrected threshold of P-value is 3.71E-06

^b^The classical Bonferroni-corrected threshold of P-value is 8.09E-05
