# Supplementary material for: Haplotype-based genome-wide association study identifies loci and candidate genes for milk yield in Holsteins
Source: PLoS One. 2018 Feb 15;13(2):e0192695. doi: 10.1371/journal.pone.0192695 (PMC5813974; doi:10.1371/journal.pone.0192695)
Supplement: S2 Table — (DOCX) [file pone.0192695.s002.docx]

S2 Table. Haplotype frequencies for each block

| Block | Chr. | Haplotype | Frequency |
| --- | --- | --- | --- |
| Block 2-1 | 1 | **H00** | **0.9483** |
|  |  | H01 | 0.0005 |
|  |  | H10 | 0.0009 |
|  |  | H11 | 0.0504 |
| Block 2-2 | 5 | **H00** | **0.8159** |
|  |  | H01 | 0.0018 |
|  |  | H10 | 0.0005 |
|  |  | H11 | 0.1818 |
| Block 2-3 | 10 | **H00** | **0.9386** |
|  |  | H01 | 0.0005 |
|  |  | H10 | 0.0005 |
|  |  | H11 | 0.0604 |
| Block 2-4 | 17 | **H00** | **0.6502** |
|  |  | H01 | 0.0009 |
|  |  | H10 | 0.0201 |
|  |  | H11 | 0.3288 |
| Block 2-5 | 19 | **H00** | **0.9336** |
|  |  | H01 | 0.0005 |
|  |  | H10 | 0.0005 |
|  |  | H11 | 0.0655 |
| Block 2-6 | 24 | **H00** | **0.6484** |
|  |  | H01 | 0.0632 |
|  |  | H10 | 0.0005 |
|  |  | H11 | 0.2880 |
| Block 2-7 | 1 | H00 | 0.0032 |
|  |  | H01 | 0.1163 |
|  |  | **H10** | **0.8800** |
|  |  | H11 | 0.0005 |
| Block 2-8 | 14 | H00 | 0.0559 |
|  |  | H01 | 0.0005 |
|  |  | **H10** | **0.7486** |
|  |  | H11 | 0.1951 |
| Block 2-9 | 20 | H00 | 0.0971 |
|  |  | H01 | 0.0005 |
|  |  | H10 | 0.0009 |
|  |  | **H11** | **0.9016** |
| Block 3-1 | 2 | **H000** | **0.7761** |
|  |  | H001 | 0.0000 |
|  |  | H010 | 0.0000 |
|  |  | H011 | 0.0000 |
|  |  | H100 | 0.0334 |
|  |  | H101 | 0.0005 |
|  |  | H110 | 0.0009 |
|  |  | H111 | 0.1891 |
| Block 3-2 | 10 | H000 | 0.0005 |
|  |  | H001 | 0.0000 |
|  |  | H010 | 0.0005 |
|  |  | **H011** | **0.9057** |
|  |  | H100 | 0.0925 |
|  |  | H101 | 0.0009 |
|  |  | H110 | 0.0000 |
|  |  | H111 | 0.0000 |
| Block 3-3 | 14 | **H000** | **0.6909** |
|  |  | H001 | 0.1282 |
|  |  | H010 | 0.0046 |
|  |  | H011 | 0.0000 |
|  |  | H100 | 0.0009 |
|  |  | H101 | 0.0000 |
|  |  | H110 | 0.1754 |
|  |  | H111 | 0.0000 |
| Block 3-4 | 19 | H000 | 0.0096 |
|  |  | H001 | 0.1571 |
|  |  | H010 | 0.0096 |
|  |  | H011 | 0.0005 |
|  |  | H100 | 0.0014 |
|  |  | H101 | 0.0005 |
|  |  | **H110** | **0.8196** |
|  |  | H111 | 0.0018 |
| Block 3-5 | 26 | H000 | 0.0522 |
|  |  | H001 | 0.0005 |
|  |  | H010 | 0.0000 |
|  |  | H011 | 0.0000 |
|  |  | H100 | 0.0000 |
|  |  | H101 | 0.0435 |
|  |  | H110 | 0.0000 |
|  |  | **H111** | **0.9038** |
| Block 3-6 | 1 | **H000** | **0.9057** |
|  |  | H001 | 0.0005 |
|  |  | H010 | 0.0000 |
|  |  | H011 | 0.0000 |
|  |  | H100 | 0.0000 |
|  |  | H101 | 0.0000 |
|  |  | H110 | 0.0005 |
|  |  | H111 | 0.0934 |
| Block 3-7 | 6 | **H000** | **0.8489** |
|  |  | H001 | 0.0014 |
|  |  | H010 | 0.0000 |
|  |  | H011 | 0.0009 |
|  |  | H100 | 0.0014 |
|  |  | H101 | 0.0005 |
|  |  | H110 | 0.0023 |
|  |  | H111 | 0.1447 |
| Block 3-8 | 17 | **H000** | **0.5852** |
|  |  | H001 | 0.3242 |
|  |  | H010 | 0.0005 |
|  |  | H011 | 0.0000 |
|  |  | H100 | 0.0005 |
|  |  | H101 | 0.0005 |
|  |  | H110 | 0.0888 |
|  |  | H111 | 0.0005 |
