# Supplementary material for: Haplotype-based genome-wide association study identifies loci and candidate genes for milk yield in Holsteins
Source: PLoS One. 2018 Feb 15;13(2):e0192695. doi: 10.1371/journal.pone.0192695 (PMC5813974; doi:10.1371/journal.pone.0192695)
Supplement: S3 Table — (DOCX) [file pone.0192695.s003.docx]

S3 Table. MAF of each SNP in significant blocks

| Chr. | Gene | SNP | MAF | Minor allele |
| --- | --- | --- | --- | --- |
| 24 | DLGAP1 | 38 309 144 | 0.288 | - |
| 24 |  | 38 309 195 | 0.351 | - |
| 19 | AP2B1 | 15 017 221 | 0.066 | - |
| 19 |  | 15 017 461 | 0.066 | - |
| 5 | ITPR2 | 83 678 733 | 0.182 | - |
| 5 |  | 83 678 739 | 0.184 | - |
| 10 | THBS4 | 10 935 068 | 0.093 | - |
| 10 |  | 10 935 325 | 0.094 | - |
| 10 |  | 10 935 342 | 0.093 | - |
| 2 | ARHGEF4 | 1 590 666 | 0.224 | - |
| 2 |  | 1 590 672 | 0.190 | - |
| 2 |  | 1 590 684 | 0.190 | - |
| 26 | TDRD1 | 34 961 904 | 0.053 | - |
| 26 |  | 34 961 905 | 0.096 | - |
| 26 |  | 34 961 908 | 0.052 | - |
| 19 | KIF19 | 57 737 460 | 0.177 | - |
| 19 |  | 57 737 480 | 0.168 | - |
| 19 |  | 57 737 777 | 0.160 | - |
| 1 | CEP63 | 135 886 514 | 0.051 | - |
| 1 |  | 135 886 546 | 0.051 | - |
| 17 | FBRSL1 | 45 596 775 | 0.349 | - |
| 17 |  | 45 596 988 | 0.330 | - |
| 10 | OR4N5 | 27 514 423 | 0.061 | - |
| 10 |  | 27 514 460 | 0.061 | - |
| 14 | OC90 | 10 142 741 | 0.176 | - |
| 14 |  | 10 142 746 | 0.180 | - |
| 14 |  | 10 142 975 | 0.128 | - |
| 1 | N/A | 29 116 305 | 0.094 | - |
| 1 |  | 29 116 327 | 0.094 | - |
| 1 |  | 29 116 348 | 0.094 | - |
| 1 | N/A | 149 340 609 | 0.120 | - |
| 1 |  | 149 340 632 | 0.117 | - |
| 6 | N/A | 57 444 519 | 0.149 | - |
| 6 |  | 57 444 521 | 0.148 | - |
| 6 |  | 57 444 587 | 0.147 | - |
| 14 | N/A | 31 436 218 | 0.056 | - |
| 14 |  | 31 436 401 | 0.196 | - |
| 17 | N/A | 66 974 027 | 0.090 | - |
| 17 |  | 66 974 043 | 0.090 | - |
| 17 |  | 66 974 064 | 0.325 | - |
| 20 | N/A | 74 328 375 | 0.098 | + |
| 20 |  | 74 328 383 | 0.098 | + |
|  |  |  |  |  |
